# The Impact of Yoyo Dieting and Resistant Starch on Weight Loss and Gut Microbiome in C57Bl/6 Mice

**DOI:** 10.3390/nu16183138

**Published:** 2024-09-17

**Authors:** Kate Phuong-Nguyen, Martin O’Hely, Greg M. Kowalski, Sean L. McGee, Kathryn Aston-Mourney, Timothy Connor, Malik Q. Mahmood, Leni R. Rivera

**Affiliations:** 1 School of Medicine, Institute for Mental and Physical Health and Clinical Translation, Deakin University, Geelong, VIC 3220, Australia; martin.ohely@deakin.edu.au (M.O.); sean.mcgee@deakin.edu.au (S.L.M.); k.astonmourney@deakin.edu.au (K.A.-M.); timothy.connor@deakin.edu.au (T.C.); 2 Metabolic Research Unit, School of Medicine, Deakin University, Waurn Ponds, VIC 3216, Australia; greg.kowalski@deakin.edu.au; 3 Murdoch Children’s Research Institute, Royal Children’s Hospital, The University of Melbourne, Parkville, VIC 3052, Australia; 4 School of Exercise and Nutrition Sciences, Institute for Physical Activity and Nutrition, Deakin University, Waurn Ponds, VIC 3216, Australia; 5 School of Medicine, Deakin University, Waurn Ponds, VIC 3216, Australia; malik.mahmood@deakin.edu.au

**Keywords:** weight cycling, yoyo dieting, obesity, resistant starch, gut microbiome, metabolism, short-chain fatty acid, fatty liver

## Abstract

Cyclic weight loss and subsequent regain after dieting and non-dieting periods, a phenomenon termed yoyo dieting, places individuals at greater risk of metabolic complications and alters gut microbiome composition. Resistant starch (RS) improves gut health and systemic metabolism. This study aimed to investigate the effect of yoyo dieting and RS on the metabolism and gut microbiome. C57BL/6 mice were assigned to 6 diets for 20 weeks, including control, high fat (HF), yoyo (alternating HF and control diets every 5 weeks), control with RS, HF with RS, and yoyo with RS. Metabolic outcomes and microbiota profiling using 16S rRNA sequencing were examined. Yoyo dieting resulted in short–term weight loss, which led to improved liver health and insulin tolerance but also a greater rate of weight gain compared to continuous HF feeding, as well as a different microbiota profile that was in an intermediate configuration between the control and HF states. Mice fed HF and yoyo diets supplemented with RS gained less weight than those fed without RS. RS supplementation in yoyo mice appeared to shift the gut microbiota composition closer to the control state. In conclusion, yoyo dieting leads to obesity relapse, and increased RS intake reduces weight gain and might help prevent rapid weight regain via gut microbiome restoration.

## 1. Introduction

Overweight and obesity have tripled worldwide since 1975, affecting more than 1.9 billion adults across all ages and socioeconomic groups. This places many people at a high risk of developing an array of comorbidities, including psychological health burdens [1] and numerous related metabolic disorders [2,3,4,5,6], such as cardiovascular diseases [7,8], type 2 diabetes [9], and fatty liver diseases [10,11]. Moreover, obesity has been associated with lowered life expectancy [12] and quality of life [13] and has become a major health and economic crisis in both developed and developing countries [14,15,16,17].

For many individuals, weight loss is difficult and often unsustainable. Current evidence suggests that 80–95% of individuals who had previously lost weight typically regained most of their lost weight within 5 years [18,19,20,21,22,23,24,25,26,27,28,29,30,31,32,33,34,35]. Repeated phases of dieting and non-dieting periods resulting in cyclic weight loss and regain are commonly called yoyo dieting [36]. Currently, weight loss remains the gold standard strategy for many metabolic complications, such as diabetes and fatty liver disease [37,38]. Yoyo dieting results in relapsing metabolic complications that can surpass the pre-weight loss metabolic derangement [39,40]. 

There has been substantial progress in studying the pathogenesis of obesity, especially with increasing evidence that gut microbiome alterations play an important role in its development. However, the role of the gut microbiome in yoyo dieting remains unknown [41,42]. Gut dysbiosis refers to an imbalance in gut microbiota homeostasis that leads to the loss of beneficial microbial organisms and changes in gut microbiota composition and function [43]. This is known to influence metabolic homeostasis and is associated with an increased risk of metabolic disturbances [44,45], including obesity, as shown in both animal [46,47,48] and human studies [49,50,51,52,53]. Moreover, it is well established that diet is one of the main drivers of the composition and function of the gut microbiome. Changes in diet profoundly impact the gut microbiome within days [54,55,56]. There is some evidence suggesting that yoyo dieting results in long–term modification of the gut microbiome profile [47]. This alteration in gut microbiota composition is believed to contribute to susceptibility to weight gain relapse, hence the challenge of maintaining weight loss [47].

Prior studies suggest that increasing the intake of resistant starch (RS), a type of dietary fibre, improves gut health [57,58,59,60,61,62,63,64] and weight management [65,66,67,68,69]. RS is a type of dietary fibre that resists digestion in the small intestine and is passed to the large intestine for fermentation by the colonic microbiome, producing short-chain fatty acids (SCFAs) [70]. These SCFAs are known to provide an energy supply for the colonic mucosa [71,72,73] and act as signalling molecules that regulate metabolic pathways [74,75,76,77,78,79,80,81,82,83,84,85]. The most abundant SCFAs are butyrate, acetate, and propionate, which are associated with reduced adiposity [86] and a decreased risk of developing cardiometabolic disease and bowel disorders [87,88,89,90]. RS can be classified into five categories based on composition and source [60,91,92,93,94,95]. This study investigated the role of RS4, which represents chemically modified starch that is commercially available as a food ingredient [95]. Our study aimed to determine the effects of yoyo dieting on gut and metabolic health and investigate whether supplementation with RS might be a promising and effective weight management strategy.

## 2. Materials and Methods

### 2.1. Animals and Diets

**Figure 1:**
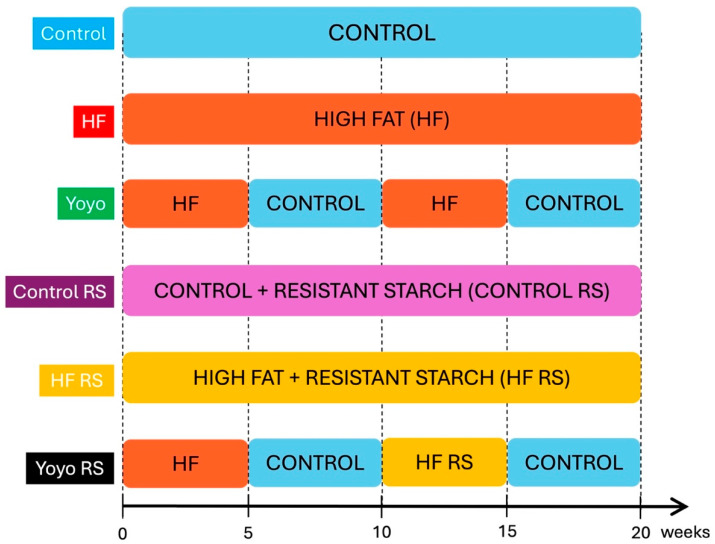
Diet treatments. Male and female mice were exposed to 6 diets for 20 weeks, including combinations of control and high-fat (HF) diets with and without resistant starch (RS).

Five-week-old male and female C57BL/6 mice were purchased from the Animal Resource Centre (Perth, WA, Australia). Following arrival at our facility, the mice were acclimatised for 1 week, during which they were fed a normal chow diet. Following this acclimation period, mice were exposed to six diet treatments (*n* = 7–8/diet group/sex) for 20 weeks. The sample size was calculated using power calculations based on previous experiments that examined changes in gastrointestinal function in obesity using a 2-tailed hypothesis with an alpha of 0.05 and a power of 0.8. Dietary groups included: (1) control (SF17-091; 12.3% kcal from fat); (2) high fat (HF, SF16-048; 60% kcal from fat); (3) yoyo, in which mice alternated between an HF and control every 5 weeks (yoyo); (4) control supplemented with RS (control RS, SF21-019, 13.9% kcal from fat); (5) HF supplemented with RS (HF RS) (SF21-018, 60% kcal from fat); and (6) yoyo supplemented with RS in the second weight gain cycle, in which mice were fed an HF (SF16-048) diet for 5 weeks, control (SF17-091) diet for 5 weeks, HF RS (SF21-018) diet for 5 weeks, and a control (SF17-091) diet for 5 weeks (yoyo RS) (Figure 1).

Six weeks of age was chosen as the starting age given the increased appearance of obesity in humans at a corresponding age (mice aged 6–26 weeks correspond to humans aged approximately 18–32 years old [96,97]). Mice were housed in groups of 4 per cage and 2 cages per diet treatment, and they were maintained on a strict 12-hour light/dark cycle, controlled temperature at 21 °C, humidity between 40–70% and given ad libitum access to irradicated pallet food and autoclaved water. 

All diets were customised by Specialty Feeds Australia (Glen Forrest, Western Australia). The RS was supplemented with GemStar^®^ RS, Hamburg, IA, USA (RS type 4, 117–140 g/kg).

There was no significant difference in the body weight of all animals at the beginning of the diet intervention. At baseline, mice weighed 20.5 ± 0.5 g (males) or 17 ± 0.5 g (females). There was no difference in food intake (in grams) between all dietary groups when measured during weeks 15–17.

All procedures were conducted according to the guidelines of the National Health and Medical Research Council and approved by the Deakin University Animal Ethics Committee. Animals were humanely culled from the study if they showed clinical illness or experienced 20% body weight loss over 7 days. 

Body Weight and Sample Collection: Body weight measurements and faecal collection were performed weekly throughout the 20-week experimental period. Mice were humanely killed by cervical dislocation after 20 weeks. Terminal cardiac blood was collected for SCFA analysis, and liver and adipose tissues (gonadal and perirenal) were collected and weighed.

### 2.2. Oral Glucose Tolerance Test

To assess glucose homeostasis under physiologically relevant conditions, an oral glucose tolerance test (OGTT) was conducted in week 19 of the study after a 5-hour fast. Glucose (2 g/kg body weight) was administrated by oral gavage in saline (200–300 µL), and tail blood was collected at 0, 15, 30, 45, 60, and 90 min post gavage for glucose level examination (AccuChek, Roche Diagnostics, Indianapolis, IN, USA) and insulin response. Insulin was determined using a mouse ultrasensitive insulin ELISA kit (ALPCO Diagnostics, Salem, NH, USA) measurement according to the manufacturer’s protocol.

### 2.3. Insulin Tolerance Test

An insulin tolerance test (ITT) was conducted in week 20 of the study. Animals were fasted for 5 h and then administrated 1.0 U/kg body weight Humulin Insulin 30/70 (Eli Lilly) via intraperitoneal injection. Blood glucose measurements were performed 0, 30, 60, 90, and 120 min following the Humulin injection (AccuChek, Roche Diagnostics, Indianapolis, IN, USA).

### 2.4. Liver Histology

Liver was fixed in 10% neutral buffered formalin, processed, paraffin-embedded, and sectioned (5 μm) before haematoxylin and eosin staining to assess steatohepatitis. Slides were examined and photographed using an EVOSTM M7000 Imaging System (AMF7000, Thermal Fisher Scientific, Waltham, MA USA). Hepatocyte ballooning was defined as regions of liver cells that had enlarged 1.5–2 times the normal hepatocyte diameter [98,99]. Hepatocyte ballooning was quantified using 20 randomised light microscopic fields per animal at ×20 magnification in a blinded fashion.

### 2.5. Liver Triglyceride Determination

Triglyceride was extracted from 20 mg of frozen mouse liver using Jouihan’s liver triglyceride assay protocol [100]. Prior to the triglyceride assay, the sample was diluted in sterile Milli-Q water with a ratio of 1/4 sample/water. The free glycerol concentration was determined in the assay using Triglycerides GPO-PAP (Cobas C pack green, Roche Diagnostics, Indianapolis, IN, USA). The hepatic triglyceride content was determined using the following equation:

Triglyceride content (mg/g of liver) = [glycerol] (mg/dL) × 0.0083 (dL)/liver mass (g)

### 2.6. Short-Chain Fatty Acids (SCFAs)

Plasma propionate and butyrate measurements were made using negative chemical ionization (NCI) gas chromatography–mass spectrometry (GC–MS, Agilent Technologies, Santa Clara, CA, USA), based on the method of Tomcik et al. [101], with some modifications described below. 

Terminal blood plasma was used for the determination of relative propionate and butyrate levels. Briefly, 40 µL of either plasma or extraction blanks (Milli-Q Water) was added to a 1 mL glass VEREXTM vial (Phenomenex, Torrance, CA, USA), followed by the addition of 60 µL of Dullbecco modified PBS (no calcium, no magnesium, pH 7.0–7.3 cell culture grade; Thermo Fisher Scientific) containing internal standards (4 µM 13C3 propionate and 4 µM 13C4 butyrate; Cambridge Isotope Laboratories, Inc., Tewksbury, MA, USA). The samples were then chemically derivatised via the addition of 200 µL of GC grade acetone containing 100 mM 2,3,4,5,6-pentafluorobenzyl bromide (PFBBr; Sigma, St. Louis, MO, USA), and after capping the samples were vortexed for 1 min and incubated at 60 °C for 1 h. Subsequently, vials were left to cool down to room temperature, and the PFB propionate and butyrate esters were extracted via the addition of 125 µL of analytical-grade isooctane, followed by 1 min of vortexing and centrifugation at 5000× *g* for 5 min. Finally, 50 µL of the top isooctane (organic) phase was removed and transferred to glass inserts held in 2 mL GC vials, ready for GC–MS analysis. The samples were analysed using an Agilent 7890B GC system and an Agilent 5977B MSD (Agilent Technologies, Santa Clara, CA, USA) in NCI mode, with helium as the carrier and methane as the reagent gas. Each sample was injected (1 μL) with a 10:1 split ratio. A VF-5 capillary column with a 10 m inert EZ-guard (J&W Scientific, Folsom, CA, USA, 30 m, 0.25 mm, 0.25 µM) was used, and the front inlet and transfer line temperatures were set to 250 °C and 270 °C, respectively, while the quadrupole and source temperatures were both set to 150 °C. The oven temperature gradient was set to 70 °C (1 min), 70 °C–160 °C at 5 °C/min, and 160–320 °C at 50 °C/min, followed by a 1-minute hold time at 320 °C. The propionate was analysed by selected monitoring of the 73 m/z (M0; unlabelled) and 76 m/z (M+3; internal standard) ions, while butyrate was analysed by selected monitoring of the 87 m/z (M0; unlabelled) and 91 m/z (M+4; internal standard) ions. All chromatographic peaks were integrated using the Quantitative Mass Hunter Workstation (Agilent Technologies, Santa Clara, CA, USA), and individual sample unlabelled-to-labelled peak area ratios were normalised to the control group, with data presented as a fold change compared to the control. 

### 2.7. Metabolic Statistical Analysis

Metabolic statistical analysis has been conducted using GraphPad Prism (version Prism 10.0.2), R (version 2023-03-15), and RStudio (version 2023.06.1+524). Descriptive and analytical statistics were performed using GraphPad Prism for multiple endpoints, such as body weight, OGTT, ITT, insulin, and SCFAs, which were analysed using two-way ANOVA (diet and RS status) with Tukey’s honestly significant difference (HSD) test for post hoc comparisons unless otherwise stated. If a two-way ANOVA for diet by RS status indicated a significant interaction between the basic diet type and RS supplementation, subsequent comparisons of individual diets were evaluated using Tukey’s HSD test. The rate of weight gain data was analysed in R and RStudio using a linear mixed effect model with the function *lmer* from the *lme4* package (version 1.1.-35.3) and the *lmerTest* package (version 3.1-3) to provide the *p*-value. Models were fitted using the REML criterium, and *p*-values came from *t*-tests using Satterthwaite’s method. This analysis was conducted to compare the average rate of body weight regain. The analysis included random and fixed effects; the random effect was the per-mouse intercept, while the fixed effects were the slope in different phases (phases 1 and 2), time points within the phase (5 weeks per phase), and diet/sex groups (4 diets, 2 sexes). Data are presented as mean ± SEM unless otherwise stated, with a *p*-value threshold of 0.05 for statistical significance. 

### 2.8. Taxonomic Microbiota Analysis

Faecal samples collected in weeks 15 and 20 were processed for microbiota analysis. DNA was isolated using QIAamp Fast DNA Stool Mini kits (Qiagen Pty Ltd., Dandenong, VIC, Australia), according to the manufacturer’s instructions. Faecal DNA was used for PCR amplification sequencing (16S rRNA producing amplicon sequence variants (ASVs), variable region V3–V4), conducted by the Australian Genome Research Facility (Melbourne, VIC, Australia). Raw data of the metagenome sequencing have been submitted to NCBI Sequence Read Archive database under submission ID SUB14694204.

Calculations of alpha diversity (Shannon and Fisher) were conducted to assess the microbiome diversity within each sample using the *estimate_richness* function in the *phyloseq* package and visualised using the *ggplot2* package. Beta diversity was measured using Bray–Curtis dissimilarities and visualised using principal coordinate analysis (PCoA) plots with *phyloseq*. To identify differences in the bacterial microbiota between groups, permutational multivariate analysis of variance (PERMANOVA) was conducted on the Bray–Curtis dissimilarity matrix with the *adonis2* function within the *vegan* package. Additionally, DESeq2 was used to determine genera that were differentially expressed (with a false discovery rate [FDR] of 0.01 to determine significance) between samples in the *phyloseq* package.

## 3. Results

### 3.1. Body Weight Change, Rate of Weight Regain, and Tissue Mass

#### 3.1.1. Body Weight Change

**Figure 2:**
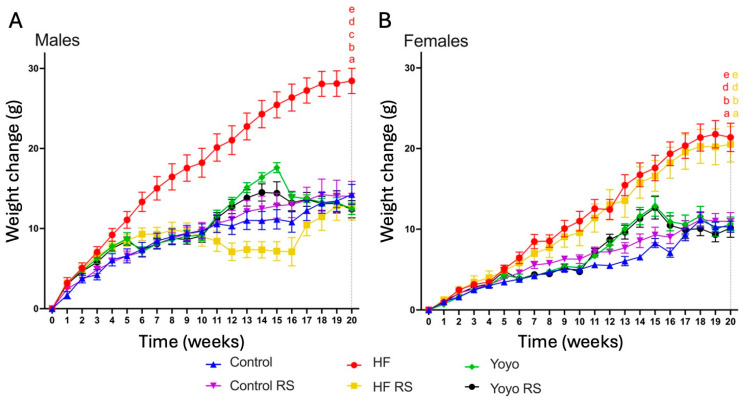
Body weight change in male (**A**) and female mice (**B**). Weight change of mice fed control (blue), HF (red), yoyo (green), control RS (purple), HF RS (yellow), and yoyo RS (black) diets. RS supplemented in an HF diet (HF RS) significantly lowered body weight change in male mice compared to an HF diet alone, but no significant difference was observed in female mice. *n* = 7–8/group/sex; statistic at week 20 *p* ≤ 0.05 versus ^a^ control, ^b^ control RS, ^c^ HF RS, ^d^ yoyo, and ^e^ yoyo RS mice. HF—high fat; RS—resistant starch.

At the end of the diet intervention, regardless of sex, yoyo mice (green) had a similar body weight change to control mice (blue) (two-way ANOVA diet × RS status in both sexes *p* < 0.0001; Tukey’s HSD: male *p* = 0.8110 (Figure 2A); female *p* = 0.9995 (Figure 2B)) and were significantly lighter than HF mice (red) (Tukey’s HSD: male *p* < 0.0001, female *p* = 0.0026 (Figure 2)).

Supplementation with RS in a control diet (control RS, purple) did not alter weight gain in male or female mice in comparison with a control diet only (Tukey’s HSD: male *p* > 0.9999 (Figure 2A), female *p* = 0.9994 (Figure 2B)). In contrast, RS supplementation in an HF diet (HF RS) (yellow) resulted in significantly less weight gain than an HF diet alone in male (Tukey’s HSD: *p* = 0.0002 (Figure 2A)) but not in female mice (Tukey’s HSD: *p* = 0.9995 (Figure 2B)).

With RS supplementation in the second phase of the yoyo diet (yoyo RS) (black), there was no significant difference in the final body weight change compared to the yoyo group (Tukey’s HSD: both sexes *p* > 0.9999 (Figure 2A,B)). Moreover, while the body weight change between yoyo RS- and HF RS-fed male mice did not differ (Tukey’s HSD: *p* > 0.9999 (Figure 2A)), the body weight change of the yoyo RS group was significantly lower than HF RS-fed female mice (Tukey’s HSD: *p* = 0.0110) (Figure 2B).

#### 3.1.2. Rates of Body Weight Gain during High-Fat Feeding Periods

**Figure 3:**
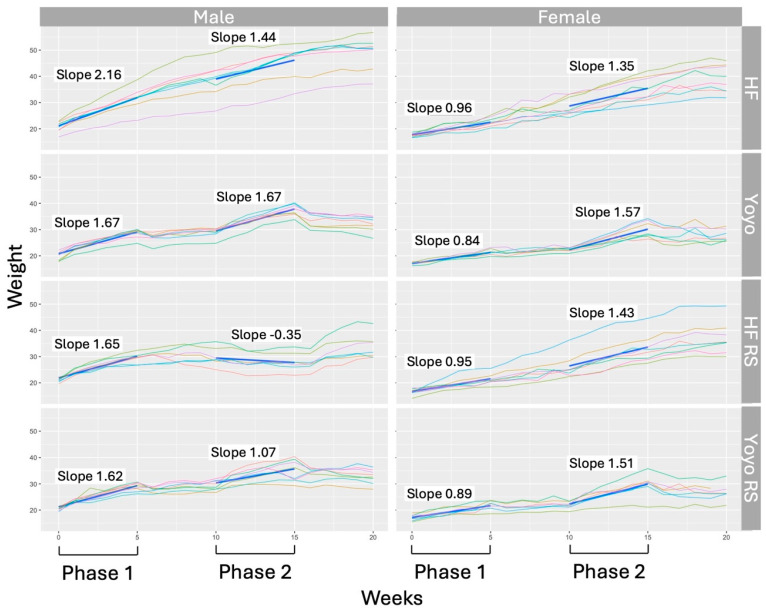
Rate of weight regain of male and female mice in high-fat and yoyo groups, supplemented with and without resistant starch, during two non-restricted (high-fat) feeding periods: Phase 1 (weeks 0–5) and Phase 2 (10–15). The weight of each animal is represented by an individual coloured line. The difference in the rate of weight gain between the 2 phases was identified as the difference in the average slopes of the same-coloured lines in Phase 1 and Phase 2. Bold blue solid lines indicate the average rate of weight gain/loss in Phase 1 and Phase 2 per diet group, respectively. In male mice, across the two HF feeding phases, yoyo mice had a significantly greater rate of weight gain compared to HF mice. RS supplementation in HF and yoyo diets (HF RS and yoyo RS) resulted in significantly lower rates of weight gain compared to diets supplemented with no RS. In female mice, across the two HF feeding phases, HF and yoyo mice had similar rates of weight gain. RS supplementation did not affect the rate of weight gain in female mice. HF—high-fat diet; RS—resistant starch.

Further analysis of body weight gain was performed on the HF and yoyo groups supplemented with and without RS to explore the rate of weight regain of yoyo mice during the two HF feeding periods: Phase 1 (weeks 0–5) and Phase 2 (weeks 10–15) (Figure 3). 

Across the two HF feeding phases, male yoyo mice had a significantly higher rate of weight gain compared to HF mice (+0.7171 g/week, *p* = 0.0087). However, in female mice, HF and yoyo mice had similar rates of weight gain (*p* = 0.2158) (Figure 3).

Supplementation with RS in an HF diet (HF RS) resulted in a significantly reduced rate of weight gain in male mice compared to those fed no RS (−1.28 g/week, *p* < 0.001). In contrast, there were no significant differences in the rates of weight gain in female mice (*p* = 0.79) (Figure 3).

With RS supplementation in the second phase of the yoyo diet (yoyo RS), male yoyo RS mice gained significantly less weight compared to yoyo mice (−1.28 g/week, *p* < 0.0001). However, the rate of weight gain of yoyo RS mice was still significantly higher than HF RS mice (+1.44 g/week, *p* < 0.001) (Figure 3). Conversely, in female mice, there was no significant difference in the rates of weight gain between the yoyo and yoyo RS groups (*p* = 0.631) and between the yoyo RS and HF RS groups (*p* = 0.6405) (Figure 3).

#### 3.1.3. Fat Mass

**Figure 4:**
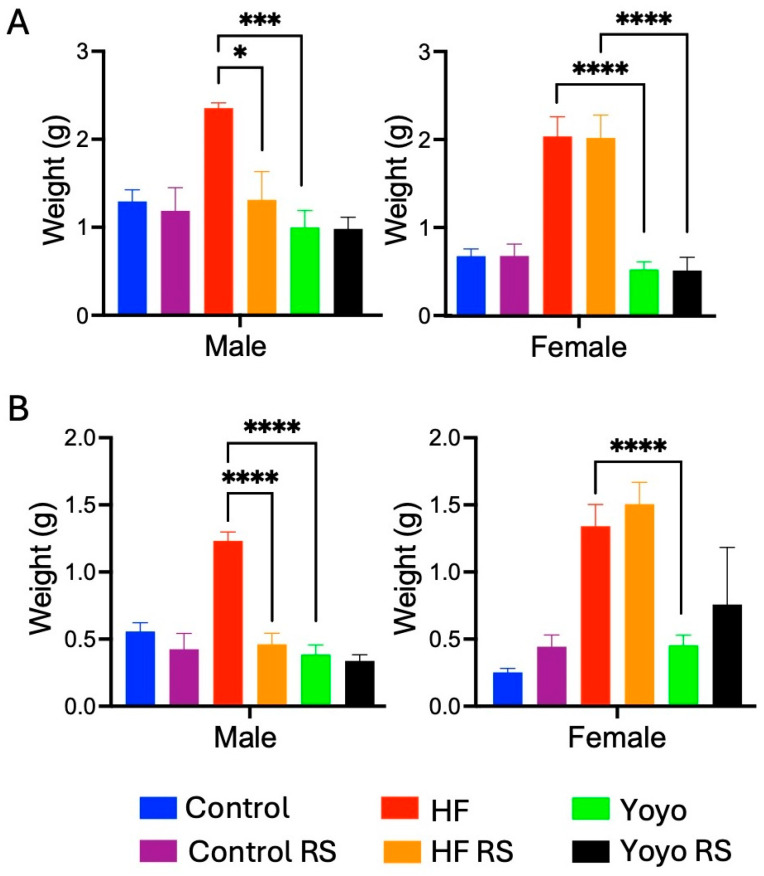
Fat mass. Gonadal fat (**A**). Perirenal fat (**B**). RS supplemented in an HF diet (HF RS) resulted in a significant reduction in fat mass in male mice compared to an HF diet alone, but no significant difference was observed in female mice. * *p* ≤ 0.05, *** *p* ≤ 0.001, **** *p* ≤ 0.0001, with *n* = 7–8/group/sex.

After 20 weeks, regardless of sex and RS status, yoyo mice (green) had similar gonadal and perirenal fat mass compared to control mice (blue) (two-way ANOVA diet × RS status: male *p* = 0.0232 and female *p* < 0.0001; Tukey’s HSD: male *p* = 0.8784 (Figure 4A) and *p* = 0.9858 (Figure 4B); female *p* = 0.9816 (Figure 4A) and *p* = 0.8352 (Figure 4B), respectively) and were significantly lower than HF mice (red) (Tukey’s HSD: male *p* = 0.0002 (Figure 4A) and *p* < 0.0001 (Figure 4B), female *p* < 0.0001 (Figure 4A) and *p* = 0.0173 (Figure 4B), respectively).

Supplementation with RS in mice fed a control diet (control RS) (purple) did not alter gonadal and perirenal fat mass compared to those fed a control diet only (Tukey’s HSD: male *p* = 0.9991 (Figure 4A) and *p* = 0.8336 (Figure 4B), female *p* > 0.9999 (Figure 4A) and *p* = 0.9716 (Figure 4B), respectively). In contrast, the supplementation of an HF diet with RS (HF RS) (yellow) resulted in significantly less gonadal and perirenal fat mass than an HF diet alone in male (Tukey’s HSD: *p* = 0.0085 (Figure 4A) and *p* < 0.0001 (Figure 4B), respectively) but not in female mice (Tukey’s HSD: *p* > 0.9999 (Figure 4A) and *p* = 0.9849 (Figure 4B), respectively).

With RS supplementation in the second phase of the yoyo diet (yoyo RS) (black), there was no significant difference in gonadal and perirenal fat mass compared to the yoyo group (Tukey’s HSD: male *p* > 0.9999 (Figure 4A) and *p* = 0.9978 (Figure 4B), female *p* > 0.9999 (Figure 4A) and *p* = 0.8847 (Figure 4B), respectively). Gonadal and perirenal fat mass between male yoyo RS mice and male HF RS mice did not differ (Tukey’s HSD: *p* = 0.8332 (Figure 4A) and *p* = 0.9469 (Figure 4B), respectively). However, gonadal fat mass in female yoyo RS mice was significantly lower than in HF RS mice (Tukey’s HSD: *p* < 0.0001 (Figure 4A).

### 3.2. Liver Health

**Figure 5:**
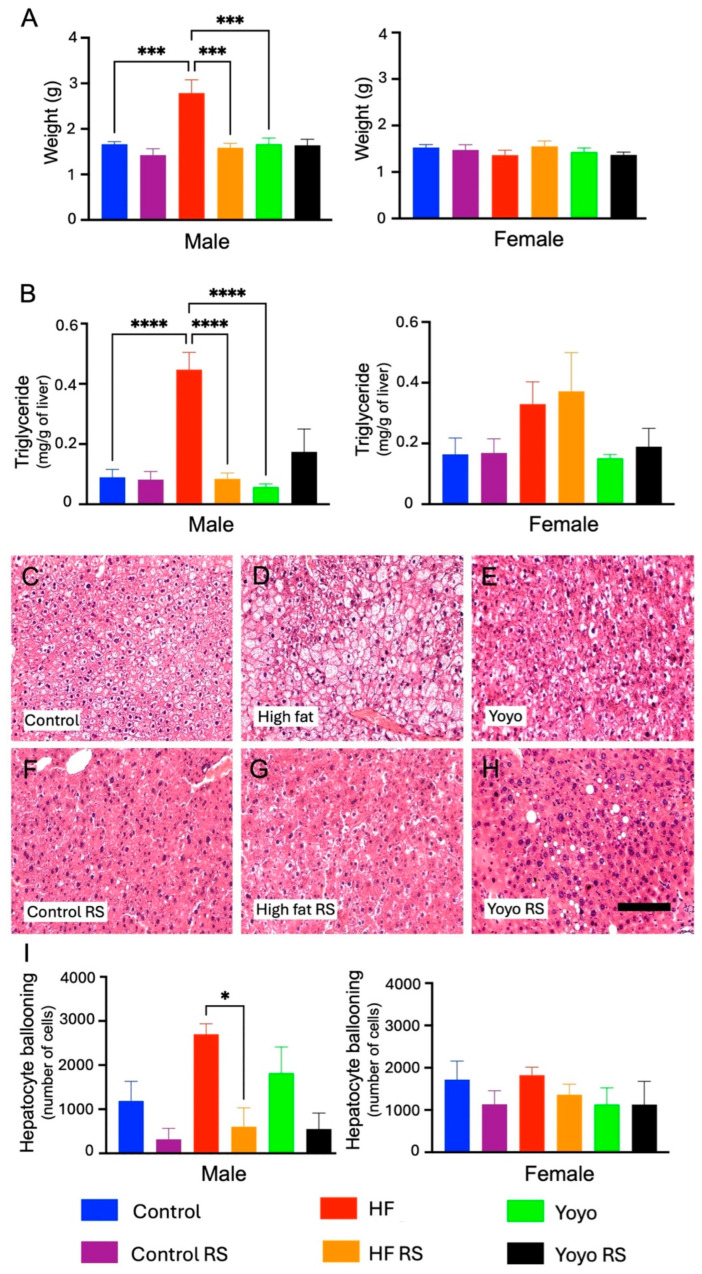
Indices of liver health. Liver mass (**A**). Liver triglycerides (**B**). Hepatocyte ballooning images of male mice using haematoxylin and eosin staining (**C**–**H**). Hepatocyte ballooning (**I**). RS supplementation in an HF diet (HF RS) significantly improved the liver health of male mice compared to an HF diet only, but no significant difference was observed in female mice. * *p* ≤ 0.05, *** *p* ≤ 0.001, **** *p* ≤ 0.0001, *n* = 7–8/group/sex. Scale bar = 150 µm. RS: resistant starch.

At the end of the dietary intervention, yoyo dieting improved indices of liver health in male mice compared to HF feeding alone. In particular, male yoyo mice (green) had similar a liver mass, liver triglycerides (two-way ANOVA diet × RS status, both *p*< 0.0001; Tukey’s HSD: both *p* > 0.9999 (Figure 5A,B)), and hepatocyte ballooning (two-way ANOVA diet × RS status, *p* = 0.0009; Tukey’s HSD: *p* = 0.8740 (Figure 5C,E,I)) compared to control mice (blue). Additionally, male yoyo mice had a significantly lower liver mass and triglycerides, but not hepatocyte ballooning, compared to HF mice (red) (Tukey’s HSD: *p* = 0.0003 (Figure 5A), *p* = 0.5401 (Figure 5B), and *p* = 0.6313 (Figure 5I), respectively). Conversely, regardless of diet and RS status, there were no differences in liver mass (two-way ANOVA diet × RS status, *p* = 0.3927 (Figure 5A)), liver triglycerides (two-way ANOVA diet × RS status, *p* = 0.9606 (Figure 5B)), or hepatocyte ballooning (two-way ANOVA diet × RS status, *p* = 0.7146 (Figure 5I)) in female mice.

Supplementation with RS in male mice fed a control diet (control RS) (purple) did not alter the liver mass (Tukey’s HSD: *p* = 0.9001 (Figure 5A)), liver triglycerides (Tukey’s HSD: *p* > 0.9999 (Figure 5B)), or hepatocyte ballooning (Tukey’s HSD: *p* = 0.6431 (Figure 5C,F,I)) compared to a control diet alone. However, supplementation with RS in an HF diet (HF RS) (yellow) resulted in a significantly lower liver mass (Tukey’s HSD: *p* = 0.0001 (Figure 5A)), liver triglycerides (Tukey’s HSD: *p* < 0.0001 (Figure 5B)), and hepatocyte ballooning (Tukey’s HSD: *p* = 0.0109 (Figure 5C,D,I)) compared to an HF diet alone.

With RS supplementation in the second phase of the yoyo diet (yoyo RS) (black), there were no significant differences in liver mass, liver triglycerides, and hepatocyte ballooning compared to the yoyo group (Tukey’s HSD: *p* > 0.9999 (Figure 5A), *p* = 0.9994 (Figure 5B), and *p* = 0.2437 (Figure 5E,H,I), respectively) and between the yoyo RS and HF RS groups (Tukey’s HSD: *p* = 0.9999 (Figure 5A), *p* > 0.9999 (Figure 5B), and *p* = 0.5663 (Figure 5G–I), respectively).

### 3.3. Blood Glucose Metabolism

**Figure 6:**
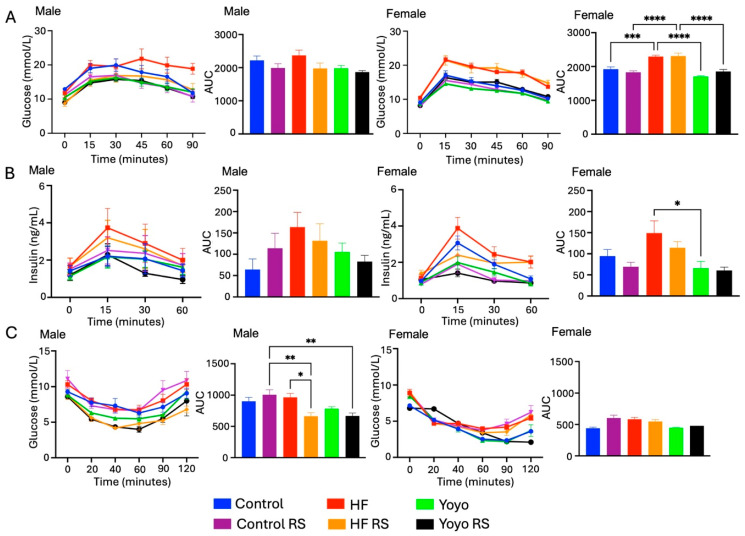
Metabolic measurements. Blood glucose during an oral glucose tolerance test (**A**). Plasma insulin during an oral glucose tolerance test (**B**). Blood glucose during an insulin tolerance test (**C**). RS supplementation in an HF diet (HF RS) resulted in a significantly improved insulin tolerance in male mice compared to an HF diet alone, but no significant difference was observed in female mice. * *p* ≤ 0.05, ** *p* ≤ 0.01, *** *p* ≤ 0.001, and **** *p* ≤ 0.0001 used to determine significance, with *n* = 7–8/group/sex. AUC: area under the curve.

In male mice, there was no significant difference in glucose and insulin levels during an OGTT, and glucose levels during an ITT between the HF (red) and control (blue) groups (two-way ANOVA diet × RS status, *p* = 0.0664 (Figure 6A), *p* = 0.1893 (Figure 6B), *p* = 0.0004 (Figure 6C); Tukey’s HSD: *p* = 0.9545, *p* = 0.1468, *p* = 0.9702, respectively) and between the yoyo and control groups (Tukey’s HSD: *p* = 0.7229, *p* = 0.8876, *p* = 0.7367, respectively). Yoyo dieting (green) appeared to improve blood glucose homeostasis compared to continuous HF feeding; however, this was not significantly different (Tukey’s HSD: *p* = 0.2536 (Figure 6A), *p* = 0.6849 (Figure 6B), *p* = 0.3299 (Figure 6C), respectively). In female mice, HF mice had significantly higher glucose tolerance (two-way ANOVA diet × RS status, *p* < 0.0001; Tukey’s HSD: *p* = 0.0006 (Figure 6A)) but similar insulin levels during an OGTT (two-way ANOVA diet × RS status, *p* = 0.004; Tukey’s HSD: *p* = 0.1906 (Figure 6B)) and glucose levels during an ITT (two-way ANOVA diet × RS status, *p* = 0.0234; Tukey’s HSD: *p* = 0.0502 (Figure 6C)) compared to control mice. There was no significant difference in glucose homeostasis and plasma insulin between the yoyo and control groups (Tukey’s HSD: *p* = 0.1312 (Figure 6A), *p* > 0.9999 (Figure 6B), *p* = 0.8399 (Figure 6C), respectively). Yoyo dieting significantly lowered glucose and insulin levels during an OGTT, but not ITT, compared to HF mice (Tukey’s HSD: *p* < 0.0001 (Figure 6A), *p* = 0.0286 (Figure 6B), *p* = 0.2294 (Figure 6C), respectively).

Supplementation with RS in mice fed a control diet (control RS) (purple) did not significantly alter glucose and insulin measured during an OGTT or insulin tolerance compared to mice fed a control diet only (male Tukey’s HSD: *p* = 0.7583 (Figure 6A), *p* = 0.8321 (Figure 6B), *p* = 0.7941 (Figure 6C), respectively; female Tukey’s HSD: *p* = 0.8731 (Figure 6A), *p* = 0.8374 (Figure 6B), *p* = 0.0528 (Figure 6C), respectively). Similarly, RS supplementation in mice fed an HF diet (HF RS) (yellow) did not significantly alter glucose and insulin levels during an OGTT compared to HF mice (male Tukey’s HSD: *p* = 0.2760 (Figure 6A), *p* = 0.9685 (Figure 6B), respectively; female Tukey’s HSD: *p* > 0.9999 (Figure 6A), *p* = 0.6550 (Figure 6B), respectively). However, male mice consuming an HF RS diet had significantly improved insulin tolerance compared to mice fed an HF diet alone (Tukey’s HSD: *p* = 0.0123 (Figure 6C)).

With RS supplementation in the second phase of the yoyo diet (yoyo RS) (black), there were no significant differences in glucose and insulin levels during an OGTT or in glucose during an ITT between yoyo RS and yoyo mice (male Tukey’s HSD: *p* = 0.9852 (Figure 6A), *p* = 0.9909 (Figure 6B), *p* = 0.7946 (Figure 6C), respectively; female Tukey’s HSD: *p* = 0.5349 (Figure 6A), *p* > 0.9999 (Figure 6B), *p* = 0.9992 (Figure 6C), respectively). Similarly, there was no difference in glucose and insulin levels in male yoyo RS and HF RS mice (Tukey’s HSD: *p* = 0.9892 (Figure 6A), *p* > 0.9999 (Figure 6B), *p* = 0.8179 (Figure 6C), respectively). On the other hand, female yoyo RS mice had significantly improved glucose tolerance compared to HF RS mice (Tukey’s HSD: *p* < 0.0001 (Figure 6A)).

### 3.4. Short-Chain Fatty Acid Concentration

**Figure 7:**
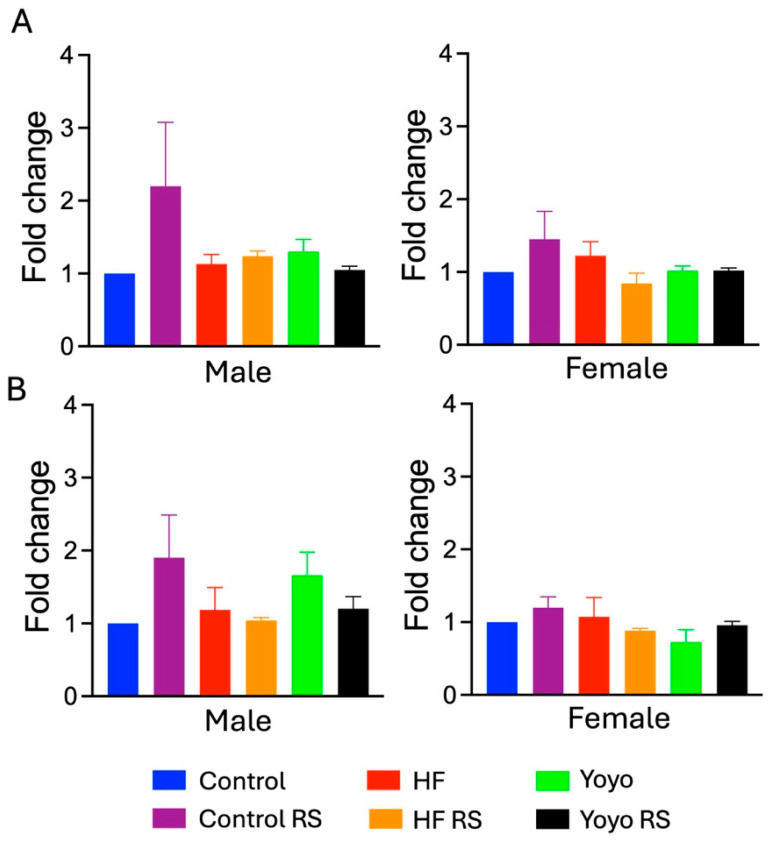
Short-chain fatty acid levels. n-Butyrate (**A**). Propionate (**B**). No change in SCFA levels across diet groups. *n* = 7–8/group/sex.

There were no significant differences in SCFA levels across all diets (propionate: two-way ANOVA diet × RS status male *p* = 0.3066, female *p* = 0.0705; n–Butyrate: two-way ANOVA diet × RS status male *p* = 0.1932, female *p* = 0.4170) (Figure 7).

### 3.5. Resistant Starch and Yoyo Dieting Reshaped Gut Microbiota

#### 3.5.1. Relative Abundance by Phylum

**Figure 8:**
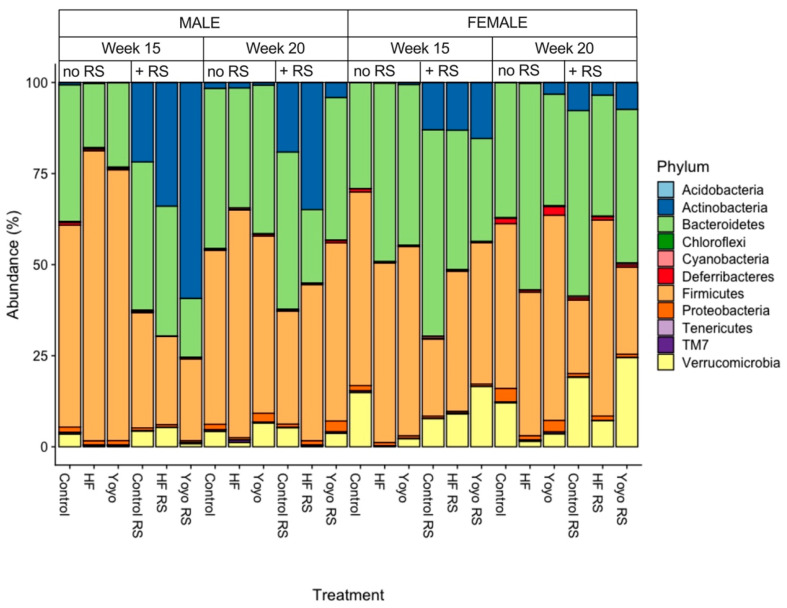
Relative abundance. Stacked column charts representing the relative abundance of amplicon sequence variants (ASVs) at the taxonomic level of the phylum. The visualisation of ASV data displays the gut microbiota of male and female mice consuming 6 different diets in weeks 15 and 20. Diets supplemented with RS were associated with a higher relative abundance of Actinobacteria. HF: high fat. RS: resistant starch.

From a simple visual inspection, in male mice, during the second phase of HF feeding, yoyo mice had enriched Bacteroidetes (23.2%) compared to HF (17.7%), but it was reduced compared to control mice (37.6%). However, the relative abundance of Firmicutes in yoyo mice (74.3%) was reduced compared to HF mice (79.7%) but enriched compared to controls (55.4%). By the end of the diet intervention, yoyo mice had lower Bacteroidetes (40.8%) and similar Firmicutes (48.6%) compared to controls (44.0% and 47.8%, respectively), but they had enriched Bacteroidetes and lower Firmicutes compared to HF mice (32.9% and 62.5%, respectively) (Figure 8, male, week 15). In female mice, during the second phase of HF feeding, yoyo mice had a lower relative abundance of Bacteroidetes (44.2%) than HF mice (49%), but it was higher than in control mice (29.1%). However, the relative abundance of Firmicutes between yoyo (52.0%), HF (49.3%), and control mice (53.2%) did not differ. By the end of the diet intervention, yoyo mice had lower Bacteroidetes (30.7%) and enriched Firmicutes (56.3%) compared to HF (56.8% and 39.4%, respectively) and control mice (37.0% and 45.2%, respectively) (Figure 8, female, week 15).

Supplementation with RS in a control diet (control RS) was associated with a greater relative abundance of Actinobacteria compared to a control diet only (Figure 8, male, week 15: 21.8% versus 0.63%, week 20: 19.09% versus 1.6%; Figure 8, female, week 15: 12.9% versus 0.05%, week 20: 7.73% versus 0.09%). Likewise, RS supplementation in an HF diet (HF RS) was also associated with enriched Actinobacteria compared to an HF diet only (Figure 8, male, week 15: 33.9% versus 0.26%, week 20: 34.9% versus 1.5%; Figure 8, female, week 15: 13.08% versus 0.15%, week 20: 3.46% versus 0.21%). 

During the second phase of HF feeding, RS supplementation in the yoyo diet (yoyo RS) resulted in a greater relative abundance of Actinobacteria compared to the yoyo diet supplemented without RS (Figure 8, male, week 15: 59.3% versus 0.1%; Figure 8, female, week 15: 15.4% versus 7.36%, respectively). Similarly, yoyo RS mice had increased Actinobacteria compared to HF RS mice (Figure 8, male, week 15: 59.3% versus 33.9%, Figure 8, female, week 15: 15.4% versus 13.08%) (Figure 8).

#### 3.5.2. Alpha Diversity

Alpha diversity indices are used to describe the microbial diversity within an ecological community with respect to its richness and/or evenness [102]. The Fisher index considers ASV richness, and the Shannon index considers both richness and evenness.

**Figure 9:**
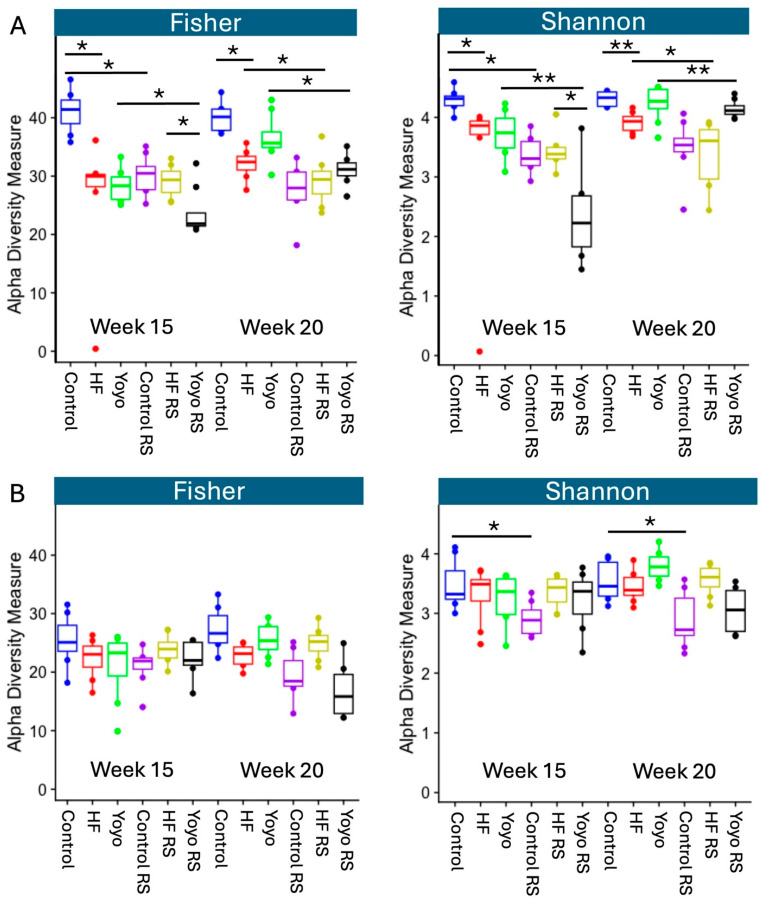
Alpha diversity. Fisher’s and Shannon’s diversity indices of faecal samples of male (**A**) and female mice (**B**) in week 15 and week 20. The median is illustrated by the horizontal line inside the box. The lowest and highest values within 1.5 times the interquartile range from the 1st and 3rd quartiles, respectively, are illustrated by whiskers. * *p* ≤ 0.05, ** *p* ≤ 0.01, with *n* = 6–8/group/sex. Boxes represent the interquartile range between the first and third quartiles. The horizontal line inside the box illustrates the median. Solid dots (●) outside the whiskers indicate greater than 1.5 times and less than 3 times the interquartile range. Graphs were generated from raw and untrimmed data. HF—high-fat diet; RS—resistant starch.

At the end of the diet intervention, male mice fed an HF diet (red) had significantly lower alpha diversity than control mice (blue) (Fisher *p* = 0.015, Shannon *p* = 0.001). Male yoyo mice (green) appeared to have an alpha diversity that was in an intermediate state between control and HF mice; however, this was not significantly different (yoyo versus control: Fisher *p* = 0.70 and Shannon *p* = 0.97; yoyo versus HF: Fisher *p* = 0.16 and Shannon *p* = 0.07 (Figure 9A, week 20)). In female mice, there were no significant differences in alpha diversity at the end of the diet intervention between the HF, yoyo, and control groups (Fisher and Shannon—yoyo versus HF: *p* = 0.36 and *p* = 0.07; yoyo versus control: *p* = 0.23 and *p* = 0.31; HF versus control: *p* = 0.91 and *p* = 0.73, respectively (Figure 9B, week 20)). 

Additionally, supplementation with RS in a control diet (control RS) (purple) significantly reduced alpha diversity in comparison with a control diet only (male: Fisher *p* = 0.005, Shannon *p* = 0.001 (Figure 9A, week 20); female: Fisher *p* = 0.11, Shannon *p* = 0.04 (Figure 9B, week 20)). Similarly, RS supplementation in an HF diet (HF RS) (yellow) reduced alpha diversity compared to an HF diet alone in male (Fisher *p* = 0.05, Shannon *p* = 0.04 (Figure 9A, week 20)) but not in female mice (Fisher *p* = 0.76, Shannon *p* = 0.97 (Figure 9B, week 20)).

Likewise, RS supplementation in the second phase of the yoyo diet (yoyo RS) (black) resulted in significantly reduced alpha diversity compared to the yoyo group in male (Fisher *p* = 0.015, Shannon *p* = 0.007 (Figure 9A, week 15)) but not in female mice (Fisher *p* = 0.91, Shannon *p* = 0.97 (Figure 9B, week 15)).

#### 3.5.3. Beta Diversity

**Figure 10:**
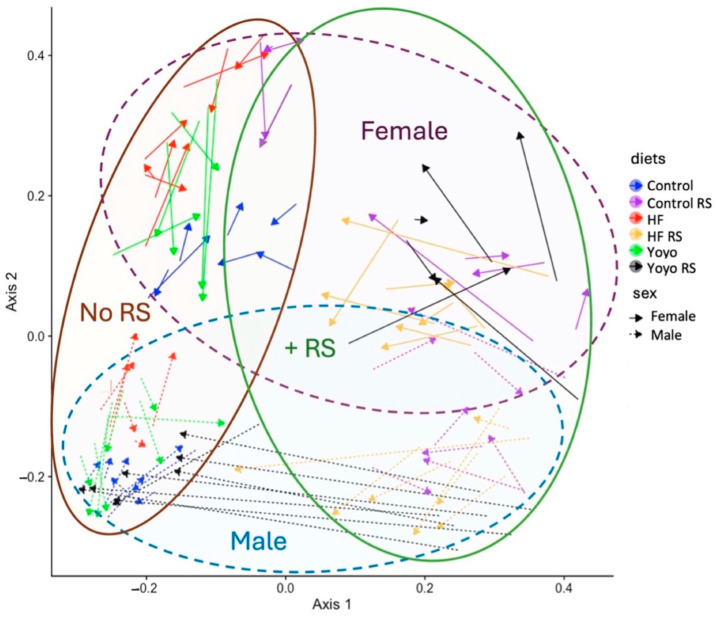
Principal coordinate analysis (PCoA)—beta diversity. Bray–Curtis ordination plot showing the dissimilarity of the gut microbiota from mice fed six different diets at two time points (weeks 15 and 20). The microbiota profiles of different dietary treatments are represented by different colours: blue (control), purple (control RS), red (HF), yellow (HF RS), green (yoyo), and black (yoyo RS). Solid arrows represent female mice, and arrows with dashed lines represent male mice. The tail of an arrow indicates microbiota composition in week 15, while the tip of an arrow indicates microbiota composition in week 20. RS—resistant starch; HF—high fat.

The Bray–Curtis measure was used to describe similarities and dissimilarities between diet treatment groups based on their gut microbial community members and abundances, and a principal coordinate analysis (PCoA) plot was constructed (Figure 10). 

HF mice (red) had a significantly different microbiota composition compared to control mice (blue) (adjusted for sex, R^2^ = 0.13, *p* = 0.001). Supplementation with RS regardless of diet (purple, yellow, black) also resulted in significantly different microbiota compositions compared to those supplemented without RS (blue, red, green) (adjusted for sex, R^2^ = 0.0650, *p* = 0.001). Moreover, we observed significantly different gut microbiota compositions in male and female mice (R^2^ = 0.0575, *p* = 0.001) (Figure 10).

At the end of the second HF feeding phase, the microbiota signatures of yoyo mice were significantly different from the control (week 15, adjusted for sex, R^2^ = 0.22, *p* = 0.001) and HF states (week 15: adjusted for sex, R^2^ = 0.05, *p* = 0.045). Moreover, by the end of the dietary intervention, this significantly altered microbiota composition of yoyo mice persisted even after a 5-week control feeding period and achieving weight loss (week 20: yoyo versus control: adjusted for sex, R^2^ = 0.09, *p* = 0.002; yoyo versus HF: adjusted for sex, R^2^ = 0.17, *p* = 0.001). Interestingly, RS supplementation in the second phase of the yoyo diet in male mice (yoyo RS) (black) appeared to exhibit a shift in the gut microbiota composition that was closer to the control group by the end of the dietary intervention. However, the microbiota profile of male yoyo RS mice remained significantly different from control mice (week 20, black versus blue, R^2^ = 0.26, *p* = 0.001). No such shift was observed in female yoyo RS mice (Figure 10).

#### 3.5.4. Differential Abundance Analysis

**Figure 11:**
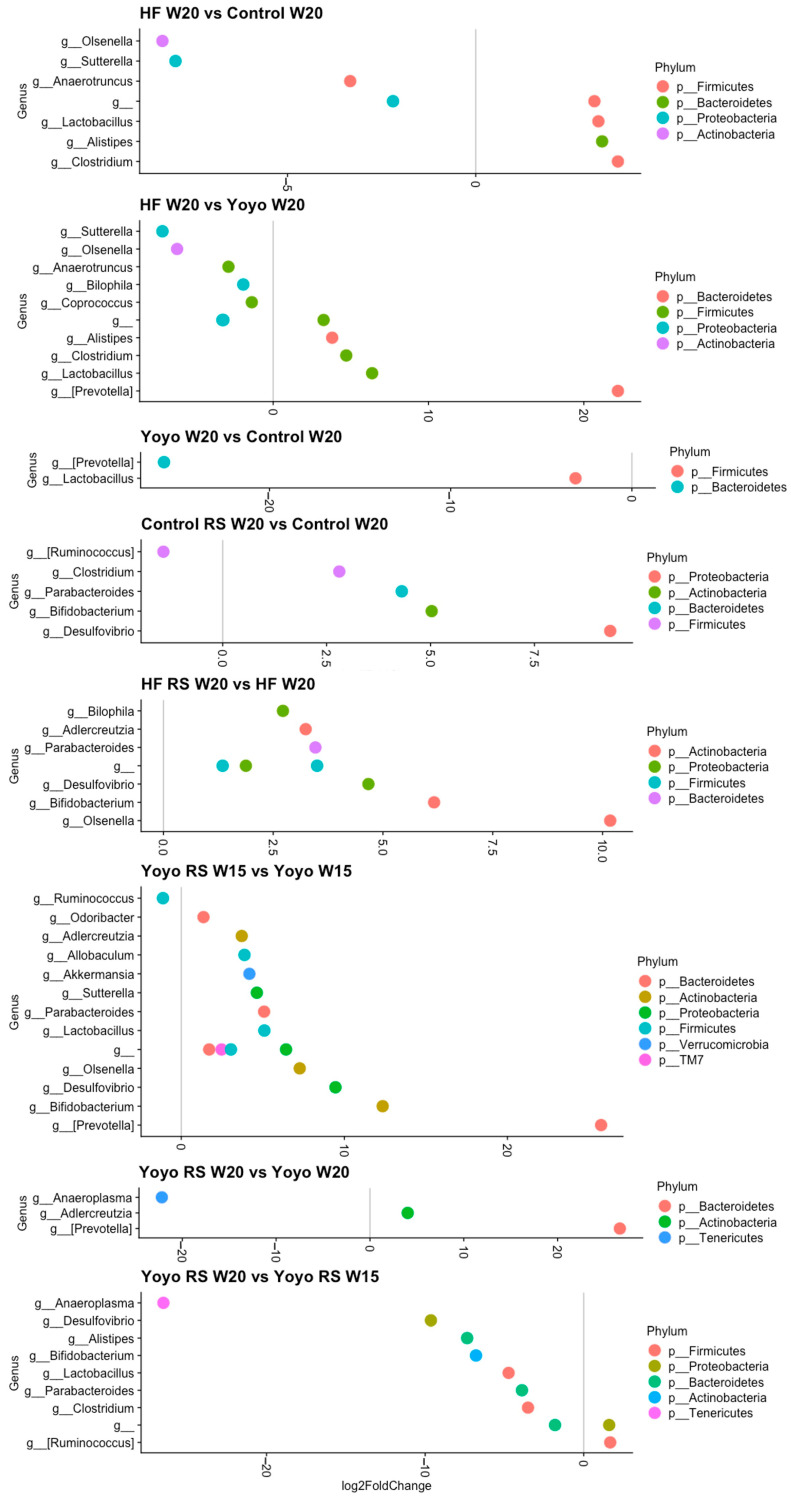
Differentially abundant genera in male mice. Only genera that were significantly different in relative abundance (FDR ≤ 0.01) are shown in the plot, with log2FoldChange estimated by DESeq2. RS—resistant starch; HF—high fat; W—week.

**Figure 12:**
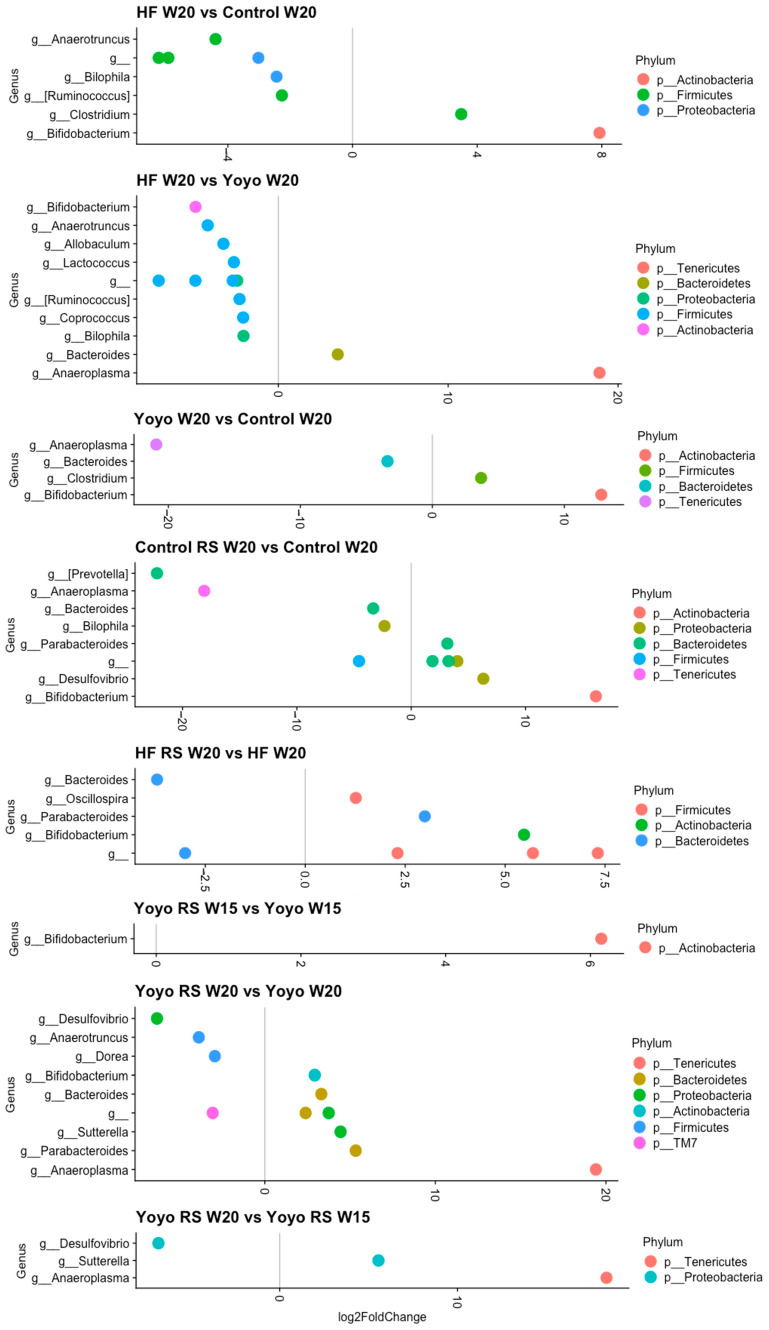
Differentially abundant genera in female mice. Only genera that were significantly different in relative abundance (FDR ≤ 0.01) are shown in the plot, with log2FoldChange estimated by DESeq2. RS—resistant starch; HF—high fat; W—week.

At the end of the diet intervention, we observed 16 (8 male; 8 female) differentially abundant genera between HF and control mice (Figure 11 and Figure 12). HF-fed mice had significantly enriched *Clostridium* (male: log2FC = 3.78; female: log2FC = 3.49) and reduced *Anaerotruncus* (male: log2FC = 3.33; female: log2FC = 4.40) compared to control mice. On the other hand, we observed 25 (12 male; 13 female) differentially abundant genera between HF and yoyo mice. HF-fed mice had significantly reduced *Anaerotruncus* (male: log2FC = −2.89; female: log2FC = −4.15), *Bilophila* (male: log2FC = −1.92; female: log2FC = −2.05), and *Coprococcus* (male: log2FC = −1.38; female: log2FC = −2.07) compared to yoyo mice. Additionally, there were six (two male; four female) differentially abundant genera between yoyo and control mice. Male yoyo mice had significantly reduced *Prevotella* (see Supplementary Materials for details on this classification) (log2FC = −25.8) and *Lactobacillus* (log2FC = −3.1) (Figure 11), while female yoyo mice had significantly reduced *Anaeroplasma* (log2FC = −20.9) and *Bacteroides* (log2FC = −3.4), but enriched *Clostridium* (log2FC = 3.7) and *Bifidobacterium* (log2FC = 12.8), compared to control mice (Figure 12).

Supplementation with RS in a control diet (control RS) resulted in 16 (5 male; 11 female) differentially abundant genera in comparison with a control diet only, particularly markedly increased *Parabacteroides* (male: log2FC = 4.31; female: log2FC = 3.16), *Bifidobacterium* (male: log2FC = 5.03 [female: log2FC = 16.16), and *Desulfovibrio* (male: log2FC = 9.32; female: log2FC = 6.31). Similarly, RS supplementation in an HF diet (HF RS) resulted in 30 (19 male; 11 female) differentially abundant genera compared to an HF diet alone, particularly enriched *Parabacteroides* (male: log2FC = 3.46; female: log2FC = 2.99) and *Bifidobacterium* (male: log2FC = 6.16; female: log2FC = 5.47) (Figure 11 and Figure 12).

In males, RS supplementation in the second phase of the yoyo diet (yoyo RS) resulted in 15 differentially abundant genera (Figure 11 and Figure 12). In particular, yoyo RS mice had enriched *Parabacteroides* (log2FC = 5.08), *Bifidobacterium* (log2FC = 12.35), *Desulfovibrio* (log2FC = 9.46), *Lactobacillus* (log2FC = 5.09), and *Adlercreutzia* (log2FC = 3.71) but reduced *Ruminococcus* (log2FC = −1.12) compared to yoyo mice. Interestingly, at the end of the dietary intervention, we only observed three differentially abundant genera in yoyo RS mice compared to yoyo mice, including *Prevotella* (log2FC = 26.63), *Anaeroplasma* (log2FC = −22.18), and *Adlercreutzia* log2FC = 4.02) (Figure 11). On the other hand, in female mice, we observed 11 differentially abundant genera between yoyo RS and yoyo mice. Particularly, yoyo RS mice had significantly enriched *Bifidobacterium* (log2FC = 6.15), *Parabacteroides* (log2FC = 5.33), and *Bacteroides* (log2FC = 3.31) but reduced *Desulfovibrio* (log2FC = −6.32) and *Dorea* (log2FC = −2.93) compared to yoyo mice (Figure 12). 

In order to explore which genera may have accounted for the shift in beta diversity closer to the control state of male yoyo RS mice, we observed nine differentially abundant genera. At the end of the dietary intervention, compared to the second phase of HF feeding with RS supplementation, yoyo RS mice exhibited significantly enriched *Ruminococcus* (also known as *Mediterraneibacter,* refer to Supplementary Materials for more details) (log2FC = 1.67) and significantly reduced *Parabacteroides* (log2FC = −3.90), *Bifidobacterium* (log2FC = −6.79), *Desulfovibrio* (log2FC = −9.64), and *Lactobacillus* (log2FC = −4.74) (Figure 11). 

## 4. Discussion

Relatively few studies have evaluated the effect of yoyo dieting and RS supplementation on metabolic and gut health. The present study revealed that yoyo dieting is both beneficial and detrimental to metabolic health, with the gut microbiome composition remaining in an intermediate state of dysbiosis. We also showed the beneficial effects of RS supplementation in improving metabolic outcomes, in addition to reshaping the gut microbiome.

### 4.1. Yoyo Dieting Appears to Be a Double-Edged Sword: Beneficial and Deleterious Effects of Yoyo Dieting on Metabolic and Gut Health

While there have been controversial findings around the effects of yoyo dieting on the metabolism, our study revealed similar metabolic outcomes following yoyo and control dieting. This aligns with previous studies [47,103] highlighting the overall importance of weight loss in improving metabolic health despite yoyo dieting. However, despite the benefits of weight loss, we also observed that yoyo dieting led to a greater rate of weight regain in male but not female mice, compared to continuous HF feeding, potentially due to gut microbial differences between sexes. This is consistent with previous studies suggesting that yoyo dieting may be a risk factor in future weight regain and obesity development [47,104,105]. Given the sex differences that we observed in metabolic health and gut microbiome changes, our study further emphasises the need to investigate both sexes in metabolic studies given the paucity of studies in this area. 

It is well established that HF diet feeding alters the gut microbiota composition [55,106,107]. There is also increasing evidence to suggest that yoyo dieting results in significantly different gut bacterial diversity and long–term alterations in gut microbiota composition [47,48]. A study by Thaiss et al. [47] investigated the long–term effects of yoyo dieting on mice exposed to two cycles of yoyo dieting. They found that despite achieving successful weight loss, the microbiota profile of yoyo mice remained in an intermediate configuration between the obese and control states. Furthermore, this post-dieting microbiome signature persisted and required a period more than five times longer than the last dieting period to reverse back to the control state. Our results are consistent with this study as we found different bacterial signatures and bacterial diversity from the control and HF states, despite successful weight loss. Collectively, these results imply that although the weight loss following yoyo dieting has short–term benefits on metabolic health [108], it is possible that the intermediate gut microbiome signature present in yoyo dieting may play a role in increasing the susceptibility to weight regain shortly after weight loss.

### 4.2. Beneficial Effects of Resistant Starch on Metabolic Health

Our findings highlight the beneficial effects of RS in improving metabolic outcomes, which are in line with numerous animal [69,109,110,111,112,113,114,115,116] and human studies [53,117,118,119,120,121]. Additionally, our study shows a striking protective effect of RS in improving metabolic outcomes, particularly in reducing body weight in male HF RS mice only. It is also important to note that there were no clinical signs of illness or changes in food intake in any of our dietary groups. The observed weight loss with RS supplementation in the HF diet (HF RS) during the second half of our study (particularly during weeks 11–16) may be due to impaired nutrient absorption and/or increased energy expenditure, which we were not able to measure in this study. Therefore, future studies are warranted to verify this hypothesis. There are also inconsistencies in the literature around RS and weight loss that could be attributed to several factors, such as variations in experimental models, treatment durations, types of RS, and different treatment dosages. Moreover, most of what we know about the effects of RS is based on animal and human studies that have mainly used RS2 (raw potato, green banana, high-amylose maize starch) [52,53,69,111,113,116,122,123,124,125,126,127,128], with limited studies on RS4 (chemically modified starch) [129,130,131,132]. Although RS2 is more commonly found in foods, it is less resistant and easily degraded by heat (such as in cooking). In contrast, RS4 offers significantly greater chemical resistance to digestive enzymes in the gut compared to RS2 [80,133].

RS represents a small portion in most foods (<2.5%, dry matter basis), even in starchy foods (<15%, dry matter basis) [91]. On average, Australian adults consume only 3–9 g of RS per day [115], which is significantly below the recommended daily intake of 15–20 g/day of RS [115] considered essential for promoting bowel health benefits [91,115]. Currently, there is no consensus on the effective dosage for different types of RS. In our study, we incorporated RS4 at fractions ranging from 11% to 14% (by weight), which is similar to previous rodent studies [134,135,136]. However, it is important to note that these doses, while effective in rodents, are not directly comparable to the RS levels required in humans. Several human studies have been conducted to explore the effective dosage of RS4 in increasing SCFA production to exert beneficial effects. A 26-week clinical study by Upadhyaya et al. [131] showed that dietary supplementation with 30% RS4 (*v*/*v* in flour) significantly increased several faecal SCFA levels in individuals with metabolic syndrome, including propionate, butyrate, valerate, and hexanoate. In another study, a dose–response trial was conducted to investigate the effectiveness of different doses of RS4 (ranging from 10 to 50 g/day of RS4) in healthy participants [132]. Their findings indicated that the majority of tested RS4 doses did not change the overall concentration of faecal SCFAs. However, a significant increase in the concentration of propionate was observed at a minimum of a very high RS4 dose of 35 g/day. SCFA concentrations in past studies were usually assessed in faecal samples as a measure of colonic absorption and metabolism. However, it is important to analyse plasma SCFA levels in order to assess their systemic effects. Therefore, in our study, we analysed plasma propionate and butyrate concentrations and observed no differences in any of our dietary groups. Of note, the concentrations of SCFAs in plasma and faecal samples are known to be different [137,138,139]. It is important to consider that SCFA concentrations can be influenced by diet, the gut microbiome, the and host metabolism [140], which can lead to considerable variations in SCFA profiles in various conditions and methods of analysis. Thus, further research is required to gain a better understanding of the role that SCFAs play in yoyo dieting and RS supplementation, as well as their potential use as biomarkers or therapeutic targets.

### 4.3. Beneficial Effects of Resistant Starch on Gut Microbiome

We observed that RS supplementation resulted in enriched *Bifidobacterium* and *Parabacteroides*, along with reduced bacterial diversity and distinct microbiota composition. The increase in the relative abundance of *Bifidobacterium* aligns with findings from various studies [79,141,142,143], showing the effect of RS in significantly altering the gut microbiome. This is likely to be advantageous as this genus is representative of microbial probiotics [144,145], which are associated with stomach acid tolerance and carbohydrate metabolism [146,147,148], anti-inflammatory properties [149], and the regulation of bowel movement [131,150,151,152]. Likewise, the enrichment of *Parabacteroides* is likely beneficial, as it has been linked to improvements in conditions such as heparinase-exacerbated acute pancreatitis [153], neuroprotection [154], and the attenuation of inflammation [155]. Notably, *Bifidobacterium* and *Parabacteroides* are both SCFA producers [153,156,157,158] that exert beneficial effects on human health [159,160]. Moreover, we observed reduced bacterial diversity following resistant starch supplementation in male mice. This was an unexpected finding as reduced diversity is often associated with dysbiosis, particularly in obesity [161,162,163]. However, the notion of diversity as an indicator of health status is being challenged, with a suggested focus towards using health-associated taxa as a better metric for health status. There is also emerging evidence showing that reduced diversity after dietary fibre intervention has been associated with improved clinical outcomes [164,165,166]. Moreover, it is important to consider that dietary fibre structure plays a key role in influencing the ability of gut bacteria to utilise these substrates [167,168]. For example, low/intermediate-specificity dietary fibres, such as RS2 [168], can be utilised and shared by many bacteria [167,168], which may promote the growth of multiple microbes, resulting in increased bacterial diversity [169]. On the other hand, RS4, a type of dietary fibre with greater specificity than RS2 [132,168], can only be utilised by a small group of bacteria [167,168], which will promote the increased growth of fewer microbes, resulting in reduced diversity [169]. This is an important consideration as reduced diversity is not always detrimental and can still be associated with improved health status.

Furthermore, we found that supplementation with RS in male yoyo mice shifted their gut microbiota profile closer to the control state following weight loss. However, the microbiota profiles of yoyo RS and control mice were still significantly different by the end of the dietary intervention, which is consistent with the findings by Thaiss et al. [47]. This suggests a promising effect of RS supplementation in shifting the gut microbiota composition in the short–term but not in the long–term restoration of the gut microbiome. Of note, microbiota restoration requires not only consuming a healthy (control) diet and achieving successful weight loss but also long–term weight management to ensure sustainable weight loss [47]. Moreover, we also observed significantly different genera associated with shifting the gut microbiome composition of yoyo RS after weight loss. These include enriched *Mediterraneibacter* and reduced *Bifidobacterium*, *Parabacteroides*, and *Desulfovibrio*, which may have contradictory implications for human health. On one hand, enriched *Mediterraneibacter* is likely not to be advantageous as this has been positively associated with increased risks of developing obesity [170], liver cancer [171], and gut dysbiosis [172]. Likewise, a reduction in *Bifidobacterium* and *Parabacteroides* might not be beneficial as they have been associated with increased risks of irritable bowel syndrome [173,174,175,176,177] and obesity [178,179,180]. However, reduced *Desulfovibrio* is likely to be beneficial as these have been associated with reduced risks of obesity [161,181,182], inflammatory bowel disease [183,184], fatty liver disease [182], and diabetes [185].

In this study, while the effect of yoyo dieting and resistant starch on the gut microbiome was investigated, one explicit limitation was the potential confounding effect of cohousing animals (four mice per cage and two cages per diet treatment). However, single housing was not feasible due to ethical concerns around compromising the biological and behavioural well-being of animals, which could result in heightened emotional stress and a lack of thermoregulation [186,187,188,189,190]. Furthermore, exploring gut microbiota changes using full-length 16S long-read sequencing or shotgun sequencing will provide valuable insights as these techniques provide higher taxonomy resolution and functional profiling.

## 5. Conclusions

In summary, our study reveals significant sex-dependent differences in metabolic outcomes and microbiome signatures. While yoyo dieting leads to metabolic health improvement through short–term weight loss, it also results in a greater risk of obesity relapse. Interestingly, increased resistant starch intake reduces body weight and may reduce the risk of rapid weight regain, potentially via gut microbiome restoration in male but not in female mice. However, the functional implications of resistant starch-induced microbiota changes for metabolic health, weight regain susceptibility, and sex differences remain to be further explored. This study emphasises the importance of improving the gut microbiome as an attractive target for sustainable weight management and the development of interventions for obesity and related conditions. Additional research is required to determine the effective dosage of resistant starch in humans to support the development of evidence-based lifestyle management initiatives to improve gut and metabolic health. Moreover, further long–term human studies are warranted to elucidate the underlying mechanisms of how resistant starch and yoyo dieting affect the gut microbiota and contribute to varying weight loss and weight regain susceptibility outcomes between sexes.

## Data Availability

The authors confirm that the data supporting the findings of this study are available within the article or upon reasonable request.

## Supplementary Materials

The following supporting information can be downloaded at: https://www.mdpi.com/article/10.3390/nu16183138/s1. Refs. [170,191,192] are cited in the supplementary materials.
