# Supplementary material for: The Impact of Yoyo Dieting and Resistant Starch on Weight Loss and Gut Microbiome in C57Bl/6 Mice
Source: Nutrients. 2024 Sep 17;16(18):3138. doi: 10.3390/nu16183138 (PMC11435396; doi:10.3390/nu16183138)
Supplement: Supplementary file 1 [file nutrients-16-03138-s001.zip › nutrients-3160213-supplementary.pdf]

nutrients

MDPI

Research Article

The Impact of Yoyo Dieting and Resistant Starch on Weight Loss and Gut Microbiome in C57Bl/6 Mice

Kate Phuong-Nguyen [1,2,*], Martin O'Hely [1,3], Greg M. Kowalski [2,4], Sean L. McGee [1,2], Kathryn Aston-Mourney [1,2], Timothy Connor [1,2], Malik Q. Mahmood [5] and Leni R. Rivera [1,2,*]

1 School of Medicine, Institute for Mental and Physical Health and Clinical Translation, Deakin University, Geelong, VIC 3220, Australia; martin.ohely@deakin.edu.au (M.O.); sean.mcgee@deakin.edu.au (S.L.M.); k.astonmourney@deakin.edu.au (K.A.-M.); timothy.connor@deakin.edu.au (T.C.)
2 Metabolic Research Unit, School of Medicine, Deakin University, Waurn Ponds, VIC 3216, Australia; greg.kowalski@deakin.edu.au
3 Murdoch Children's Research Institute, Royal Children's Hospital, The University of Melbourne, Parkville, VIC 3052, Australia
4 School of Exercise and Nutrition Sciences, Institute for Physical Activity and Nutrition, Deakin University, Waurn Ponds, VIC 3216, Australia
5 School of Medicine, Deakin University,Waurn Ponds, VIC 3216, Australia; malik.mahmood@deakin.edu.au
* Correspondence: nguyenkate@deakin.edu.au (K.P.-N.); leni.rivera@deakin.edu.au (L.R.R.)

Supplementary Materials: The following supporting information refers to section 3.5.4, "Differential Abundance Analysis".

Prevotella: An NCBI BLAST search found matches for the relevant ASVs to a variety of *Alloprevotella sp.*, *Prevotella sp.* and *Prevotellamassilia sp.* sequences. *Alloprevotella* [1] included a species previously classified as *Prevotella*, and *Prevotellamassilia sp.* [2] contains a single novel species and is presently not validly published.

Ruminococcus has been identified in our pipeline. All such ASVs were identified as "[*Ruminococcus*] *gnavus*", and *Ruminococcus gnavus* has recently been reclassified as *Mediterraneibacter gnavus* [3].

References

1. Downes, J., et al., *Description of Alloprevotella rava gen. nov., sp. nov., isolated from the human oral cavity, and reclassification of Prevotella tannerae Moore et al. 1994 as Alloprevotella tannerae gen. nov., comb. nov.* International Journal of Systematic and Evolutionary Microbiology, 2013. **63**(Pt_4): p. 1214-1218.
2. Ndongo, S., et al., *"Prevotellamassilia timonensis," a new bacterial species isolated from the human gut.* New Microbes and New Infections, 2016. **13**: p. 102-103.
3. Togo, A.H., et al., *Description of Mediterraneibacter massiliensis, gen. nov., sp. nov., a new genus isolated from the gut microbiota of an obese patient and reclassification of Ruminococcus faecis, Ruminococcus lactaris, Ruminococcus torques, Ruminococcus gnavus and Clostridium glycyrrhizinilyticum as Mediterraneibacter faecis comb. nov., Mediterraneibacter lactaris comb. nov., Mediterraneibacter torques comb. nov., Mediterraneibacter gnavus comb. nov. and Mediterraneibacter glycyrrhizinilyticus comb. nov.* Antonie van Leeuwenhoek, 2018. **111**(11): p. 2107-2128.
